# The Mechanosensory Role of Osteocytes and Implications for Bone Health and Disease States

**DOI:** 10.3389/fcell.2021.770143

**Published:** 2022-02-21

**Authors:** Jung Un Ally Choi, Amanda W. Kijas, Jan Lauko, Alan E. Rowan

**Affiliations:** Australian Institute for Bioengineering and Nanotechnology, The University of Queensland, Brisbane, QLD, Australia

**Keywords:** bone homeostasis, osteocytes, integrins, mechanotransduction, signaling pathway, aging, osteoporosis, bone therapeutics

## Abstract

Bone homeostasis is a dynamic equilibrium between bone-forming osteoblasts and bone-resorbing osteoclasts. This process is primarily controlled by the most abundant and mechanosensitive bone cells, osteocytes, that reside individually, within chambers of porous hydroxyapatite bone matrix. Recent studies have unveiled additional functional roles for osteocytes in directly contributing to local matrix regulation as well as systemic roles through endocrine functions by communicating with distant organs such as the kidney. Osteocyte function is governed largely by both biochemical signaling and the mechanical stimuli exerted on bone. Mechanical stimulation is required to maintain bone health whilst aging and reduced level of loading are known to result in bone loss. To date, both *in vivo* and *in vitro* approaches have been established to answer important questions such as the effect of mechanical stimuli, the mechanosensors involved, and the mechanosensitive signaling pathways in osteocytes. However, our understanding of osteocyte mechanotransduction has been limited due to the technical challenges of working with these cells since they are individually embedded within the hard hydroxyapatite bone matrix. This review highlights the current knowledge of the osteocyte functional role in maintaining bone health and the key regulatory pathways of these mechanosensitive cells. Finally, we elaborate on the current therapeutic opportunities offered by existing treatments and the potential for targeting osteocyte-directed signaling.

## Introduction

The most long-lived bone cells, osteocytes are known as the master regulator of bone formation and resorption (Bonewald 2011). The mechanosensory role of osteocytes underlies well-balanced bone homeostasis, which is primarily influenced by matrix strain and fluid shear stress (Weinbaum et al., 1994; Han et al., 2004; Robling and Turner 2009; Wittkowske et al., 2016). Through mechanotransduction processes, osteocytes are able to transduce extracellular signals to elicit cellular responses by initiating different signaling pathways accordingly to bring functional responses. The dysregulation of osteocyte behavior can lead to reduced bone mass and bone fragility observed in osteoporotic patients. For this reason, osteocyte-induced mechanotransduction has been studied extensively, however, the exact mechanisms and signaling pathways are not fully understood. Here we highlight the crucial role of osteocytes in bone homeostasis, including regulation of the overall bone remodeling process, perilacunar/canalicular remodeling, and systemic regulatory roles on other tissues such as the kidney, parathyroid, and heart (Dallas et al., 2013; Creecy et al., 2021). Furthermore, we summarize the osteocyte mechanosensors and the current state of mechanoresponsive signaling pathways identified in osteocytes with therapeutic implications. To achieve this, we present both *in vivo* and *in vitro* approaches that have been employed to understand the complex regulatory processes that underly osteocytes’ quintessential mechanosensory role in bone.

## Bone Homeostasis

Bone is a weight-bearing tissue, which supports locomotion, protects soft tissue, and is also known as a reservoir for calcium and phosphate (Bellido 2014; Florencio-Silva et al., 2015). Bones are composed of both organic matrix, comprising of largely type I collagen (90%), with the remaining protein component including osteocalcin, osteonectin, osteopontin, fibronectin, and thrombospondin-2 (Sroga and Vashishth 2012), as well as inorganic matrix minerals, mainly comprised of hydroxyapatite (Ca_5_(PO_4_)_3_OH) but also including small amounts of potassium, magnesium, sodium, strontium, and calcium salts (Lin et al., 2020). The formation of the bone matrix is initiated by the collagen assembly followed by hydroxyapatite deposition and tuned by minerals and amino acids of non-collagenous proteins (Young 2003; Tavafoghi and Cerruti 2016). The balance of mineral content is important and directly relates to mechanical strength (Faibish et al., 2006). For example, bones become brittle when mineral content is too high and less load-bearing if the mineral content is too low. This mineralized tissue undergoes remodeling to maintain its integrity and is tightly regulated by a precise balance of bone formation and resorption under the control of local and systemic factors, such as cytokines, hormones, and mechanical stimulation (Florencio-Silva et al., 2015). This complex process is a cycle of localized bone resorption to remove old or damaged bone followed by a longer phase of bone formation, both in an equilibrium to maintain healthy bone. The imbalance of this regulation often leads to bone diseases such as osteoporosis and is caused by a variety of factors such as aging, menopause, drugs, and changes in physical activity (Feng and McDonald 2011). Imbalance can also stem from genetic mutations, leading to bone overgrowth disorders due to defective signaling pathways that change the equilibrium resulting in van Buchem disease and sclerosteosis (Balemans et al., 1999; Sebastian and Loots 2018).

Bone is a rigid and load-bearing tissue designed to sustain high mechanical loads during exercise (Benedetti et al., 2018). There are two types of bone tissue, the cortical and trabecular bone, which have the same cells and matrix but differ in structural-functional roles (Clarke 2008). Cortical bones are more calcified and hard, and carry out the role of providing mechanical stability and form a protective layer for the internal cavity (Boskey and Coleman 2010). In comparison, the trabecular bones only contain 1/3 of calcified bone compared to the cortical ones and are mainly involved in metabolic as well as biomechanical functions (Clarke 2008). The composition of the bone matrix is important for fracture resistance, which largely depends on the geometric (size and shape) and material properties (mineral content and composition) (Osterhoff et al., 2016). Bones constantly experience mechanical forces created by various stimuli including fluid flow shear stress, hydrostatic pressure, and direct cellular deformation induced by gravitational forces as a weight-bearing tissue and loading-induced stimuli such as compressive force. The calcified bone matrix may induce micro-deformation with a maximum of 3% strain changes (Hart et al., 2017). The matrix deformation during locomotion is between 0.04 and 0.3% but hardly exceeds 0.1%. Surprisingly *in vitro* studies need to apply more than 10 times this mechanical stimulation to observe osteocyte responses, otherwise, the strain amplification at the cellular level is too small to initiate mechanotransduction pathways (Rubin and Lanyon 1984; Fritton et al., 2000). These *in vitro* forces applied back at the tissue level would cause a fracture (Burr et al., 1996; You et al., 2000; Wang et al., 2015). This difference has to be taken into consideration to more accurately capture the differences between *in vivo* and *in vitro* systems and facilitate the accurate interpretation of a translational approach. The externally applied force is transduced by highly mechanosensitive osteocytes that coordinate the effector cells, bone-forming osteoblasts, and bone-resorbing osteoclasts demonstrating the skeletal adaptation response of mechanical cues into biochemical signals (Bonewald 2011; Schaffler and Kennedy 2012). The above highlights the need to understand the mechanisms underlying osteocyte’s important regulatory role in bone homeostasis.

## Osteocytes, the Master Regulator

Osteocytes are the most abundant (∼95%) bone cells, which reside in the hard bone matrix (Hellmich and Ulm 2002; Bonewald 2011). Osteocytes are a terminally differentiated post-mitotic cell type from the osteogenic lineage, derived from mesenchymal stem cell progenitors (Pittenger et al., 1999; Day et al., 2005; Gaur et al., 2005; Sudo et al., 2007). Mesenchymal stem cell differentiation leads to osteoblasts, and a subpopulation is known to terminally differentiate into osteocytes that are individually embedded within small chambers called lacunae (Palumbo et al., 1990; Candeliere et al., 2001). After the differentiation process, the most striking morphological change of mature osteocytes is the development of unique dendritic cell processes. These dendritic cell processes create an extensive cellular network in the hard bone matrix, which enables osteocytes to communicate with neighboring osteocytes, and the osteoblasts and osteoclasts on the bone surface by creating a neuron-like network (Palumbo et al., 1990). This highly complex communication network, is created through a space called canaliculi, which are narrow channels in the hydroxyapatite matrix. Osteocytes are separated from the mineralized bone matrix, by a pericellular space filled with proteoglycan-rich matrix (glycocalyx) and interstitial fluid (Termine et al., 1981; Sauren et al., 1992; Aarden et al., 1996). Based on the measurements and mathematical models for branching, these dendritic processes were estimated to form approximately 23 trillion connections and span a total length of over 175000 km within the human body (Buenzli and Sims 2015). *In vivo*, each cell has been shown to have a varying number of dendritic cell processes ranging from 18 to 106, which reduces with aging (Beno et al., 2006; Qin et al., 2020). Initial development of dendritic cell processes from the cell body leads to the formation of subsequent subbranches, which create sufficient surface area for efficient communication with other cells and also serving as a mechanosensory structures. The unique environment for osteocytes is called the lacunocanalicular network (LCN) and is a complicated network within bone tissue with a total surface area of approximately 215 m^2^ (Sims 2016; Martin 2019) and is also thought to provide a route for the provision of nutrients, oxygen, and biochemical signals.

Since osteocytes reside in this unique LCN architecture of mineralized matrix, it has been a challenge to study osteocytes. In spite of this challenge, a variety of mechanical stimulations such as fluid flow shear stress, and substrate deformation are known to influence osteocytes functions (You et al., 2000; McGarry et a., 2005; Wittkowske et al., 2016; Qin et al., 2020). It is important to understand that loading-induced matrix deformation not only changes fluid flow velocity, but also the matrix strain, which is closely associated with the LCN architecture. A recent study demonstrated that the LCN architecture is a key determinant for bone adaption in response to mechanical stimulation (van Tol et al., 2020). The fluid flow-induced velocity was strongly dependent on the LCN architecture in a highly dense and connected network (Denisov-Nikol’skiĭ Iu and Doktorov, 1987; Johnson 1984). Another study observed that the fluid flow velocity was not directly correlated to the loading-induced strain deformation, but more associated with the LCN structure based on *in vivo* mice micro-computed tomography (microCT) evaluations (van Tol et al., 2020). The changes in the strain distributions of the LCN upon applying various static and cyclic loads highlighted the diversity of mechanosensors on these cells and complexity of the underlying mechanotransduction pathways (Wang et al., 2015). The dendritic morphology of osteocytes itself is also proposed to be synergistic with the highly-dense LCN network, resulting in an actin-rich cytoskeleton, which enables cell–cell communications between osteocytes allowing a cascade of intracellular events capable of generating a functional response (Burra et al., 2010; Hemmatian et al., 2017).

## Osteocyte Functions in Both Bone and Extraskeletal Roles

**FIGURE 1:**
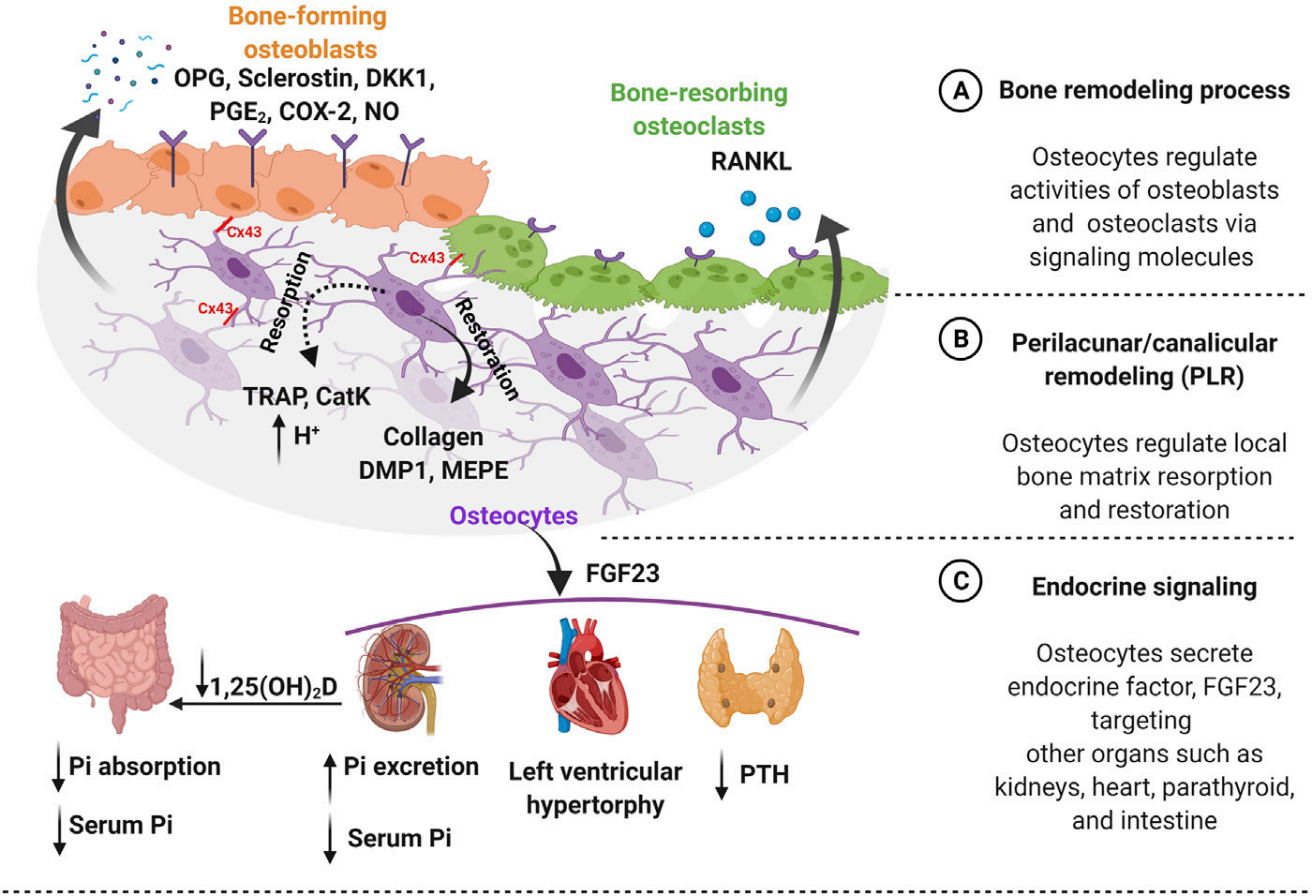
Osteocytes function in both bone homeostasis and endocrine signaling. **(A)** Bone homeostasis is guided by osteocytes, which require a precise balance of bone formation and bone resorption. Osteocytes regulate this dynamic equilibrium by releasing signaling molecules such as osteoprotegerin (OPG), sclerostin, dickkopf-related protein 1 (DKK1), prostaglandin E2 (PGE_2_), cyclooxygenase-2 (COX-2), and nitric oxide (NO) for bone-forming osteoblasts. Furthermore, osteocytes secrete receptor activator of nuclear factor-κB ligand (RANKL) for bone-resorbing osteoclasts on the bone surface. Additionally, osteocytes mediate communication with osteoblast and osteoclast via connexin 43 (Cx43) gap junctions. **(B)** Osteocytes regulate the local bone matrix through a process called perilacunar/canalicular remodeling (PLR). Matrix resorption (dotted line) is initiated creating an acidic environment by osteocyte-derived enzymes, such as tartrate-resistant acid phosphatase (TRAP) and cathepsin K (CatK) followed by matrix restoration (solid line), by producing collagen and bone matrix proteins such as dentin matrix protein 1 (DMP1) and matrix extracellular phosphoglycoprotein (MEPE). **(C)** Osteocytes secrete an endocrine factor - fibroblast growth factor (FGF23) to target other organs such as kidneys, heart, and parathyroid. The FGF23 hormone triggers parathyroid to reduce the level of parathyroid hormone (PTH). Moreover, FGF23 increases the risk of heart failure such as left ventricular hypertrophy. Importantly, FGF23 regulates serum phosphate (Pi) level by targeting kidneys by increasing phosphate excretion and also inhibiting the conversion of active vitamin D to 1,25-dihydroxy vitamin D [1,25(OH)_2_D] in the intestine to decrease phosphate resorption leading to lower serum phosphate level. Figure created using BioRender.

As osteocytes are embedded individually within the bone and separated from the effector bone cell types, they utilize secreted signaling molecules to communicate their “instructions” in addition to their broader systemic effects (Dallas et al., 2013; Prideaux et al., 2016). Osteocytes are involved in the secretion of signaling molecules, to regulate osteoblast and osteoclast activities, as well as establishing direct physical connections *via* gap junctions (Figure 1A). The quintessential molecule in bone regulation is the osteocyte-specific sclerostin, which is exclusively expressed by mature osteocytes. Sclerostin is an anti-bone formation (antagonist) regulator that directly inhibits the proliferation and differentiation of osteoblasts (Duan and Bonewald 2016). The osteoblast-induced bone formation is initiated by secreted Wnt ligand glycoproteins, which bind to low-density lipoprotein receptors (LRP) 4/5/6 for phosphorylation, leading to suppression of glycogen synthase kinase 3 (GSK3) (Karner and Long 2017). This stabilizes β-catenin, which then translocates into the nucleus, and acts as a transcriptional co-activator. Sclerostin, the protein product of the *SOST* gene expressed by mature osteocytes, binds to the LRP 4/5/6 to inhibit Wnt-binding for the Wnt/β-catenin signaling pathway in osteoblasts. Sclerostin expression is also known to be decreased by mechanical loading and increased in response to unloading conditions such as microgravity and reduced physical levels in bed-ridden patients (Pajevic et al., 2013; Bradbury et al., 2020).

The osteocyte-secreted dickkopf-related protein 1 (DKK1) binds to LRP4/5/6 on osteoblasts and acts as a Wnt competitive inhibitor (Li et al., 2006). Osteocytes also secrete osteoprotegerin (OPG), a soluble decoy receptor for receptor activator of nuclear factor-κB ligand (RANKL), which is a cytokine that binds to osteoclasts, promoting bone resorption (Kearns, Khosla, and Kostenuik 2008). The ratio between OPG and RANKL is commonly used as an indicator of bone mass and decreased RANKL/OPG ratio was reported in response to mechanical stimulation, leading to reduced osteoclast activity (Goldring 2015). Interestingly, proinflammatory cytokines were also observed to be down-regulated by mechanical loading, except interleukin 6 (IL-6) (Pathak et al., 2015; Pathak et al., 2020). Mechanical stimulation modulates the release of other factors such as nitric oxide (NO), prostaglandin E2 (PGE_2_), cyclooxygenase-2 (COX-2), and adenosine triphosphate (ATP) in osteocytes (Li et al., 2021). Especially, loading-induced calcium ions (Ca^2+^) oscillation releases signaling molecules such as NO, PGE_2_, insulin-like growth factor-1 (IGF-1), and β-catenin, which are important for osteocyte viability and anabolic effect on bone (Morrell et al., 2018).

Osteocytes, embedded in the hard bone matrix are also known to regulate their local microenvironment through a process called perilacunar/canalicular remodeling (PLR) (Figure 1B) (Qing and Bonewald 2009; Lin et al., 2020). Earlier studies observed the enlarged lacunae during lactation to release calcium from a mineralized matrix for high calcium demand situations and also in pathological conditions such as Paget’s disease, a bone loss disorder, suggesting the removal of perilacunar matrix by osteocytic osteolysis (Zambonin Zallone et al., 1982; Teti and Zallone 2009; Tsourdi et al., 2018). However, this microenvironment remodeling is also known as a homeostatic mechanism to maintain the perilacunar/canalicular network under healthy conditions such as lactation (Dole et al., 2017). Interestingly, osteocytes are able to remove both minerals and collagen from their surrounding perilacunar matrix by upregulating the H^+^ proton pump, creating an acidic microenvironment (Qing et al., 2012; Creecy et al., 2021). The acidic environment can be induced by the parathyroid hormone (PTH) upregulation during lactation (Jähn et al., 2017). Osteocyte-derived matrix removal was observed in lactating mice showing the enlarged lacunar area with upregulation of tartrate-resistant acid phosphatase (TRAP) and cathepsin K (CatK, encoded by the *Ctsk* gene), which were previously thought to be osteoclast-specific (Nakano et al., 2004; Qing et al., 2012; Lotinun et al., 2019). TRAP is an enzyme that is responsible for the dephosphorylation of bone matrix phosphoproteins and CatK is a lysosomal cysteine protease that contains the catalytic mechanism necessary for bone matrix degradation leading to bone resorption (Dai et al., 2020). By increasing osteoclast-like markers, they create an acidic environment via carbonic anhydrase 2 (Car2) and proton-pumping vacuolar ATPases.

Recent research hinted that transforming growth factor beta (TGF-β) is possibly associated with the PLR process (Schurman et al., 2021). *In vitro* studies have demonstrated TGF-β treatment upregulated *Ctsk* and matrix metalloproteinase 14 gene expressions in both osteocytic cell lines, MLO-Y4 and Ocy454 (Dole et al., 2017; Kegelman et al., 2020). Furthermore, intracellular pH (pHi) has been shown to decrease after TGF-β treatment, resulting in cell acidification, inducing PLR resorption that was dependent on the TGF-β receptors on osteocytes (Dole et al., 2017). TGF-β intake by osteocytes was blocked by using type I TGF-β receptor (TβRI) inhibitor (SB-431542), and lead to increased pH levels, equivalent to untreated groups. *In vivo* studies using osteocyte-specific TGF-β receptor knockout mice showed decreased expression of *Ctsk* leading to decreased bone resorption contributing to increased bone mass in these animals. (Dole et al., 2017; Schurman et al., 2021). Furthermore, TGF-β treatment induced changes in gene expression levels of sclerostin in osteocytes. PLR was also induced in osteocytes after recombinant human sclerostin (rhSCL) treatment, which lowers the pHi showing upregulation of catalytic genes (e.g., *Ctsk*, *Car2*, *TRAP*) (Kogawa et al., 2013). This suggests that osteocyte-produced sclerostin promotes catalytic activity to release the minerals. Moreover, rhSCL treatment in human trabecular bone samples showed an increased lacunar area around osteocytes (Kogawa et al., 2018). The activity of sclerostin was also confirmed by Lrp4/5/6 receptors, known for sclerostin binding inhibited osteocyte-mediated catalytic activity for the removal of bone matrix in PLR. However, further investigation is required to understand the mechanisms of PLR, which is different from osteoclast-mediated bone resorption.

It is well known that the increased lacunar area returns to normal after the weaning process suggesting osteocytes play a role in local matrix restoration. It was proposed, that the local PLR remodeling process was independent of mechanical stimulation and was presumed to be hormonally regulated (Qing et al., 2012; Bach-Gansmo et al., 2016). However, a recent study of mice under microgravity conditions, which removes mechanical loading on bones, observed enlarged lacunae size and deformed bone microstructure in these animals (Gerbaix et al., 2017). For osteocytes to perform PLR, both collagen production and mineralization are essential for the matrix restoration process. Previous studies support the osteocyte-driven collagen production using novel GFP-collagen transgenic mice (Baylink and Wergedal 1971; Zambonin Zallone et al., 1982; Kamel-ElSayed et al., 2015). They observed bright collagen production around some osteocytes suggesting heterogeneity in the osteocyte population has a capability of collagen production for PLR restoration. The fact that the collagenous matrix aligned with the axis of the lacunae, suggests collagen orientation and alignment are also coordinated by osteocytes. During this process, the levels of bone matrix proteins such as dentin matrix protein 1 (DMP1) and matrix extracellular phosphoglycoprotein (MEPE) in osteocytes were highly up-regulated to support the mineralization process (Gluhak-Heinrich et al., 2007; Harris et al., 2007; Teti and Zallone 2009; Qing et al., 2012).

PLR contributes to bone quality by altering the mineral to matrix ratio (M/M ratio), which is often used to predict the biomechanical properties of bone (Takata et al., 2011). The M/M ratio often increases with elevated bone mineral density contributing to better bone quality and is significantly increased with exercise (Kohn et al., 2009; Gardinier et al., 2016). *In vivo* studies of mice undertaking treadmill running, showed an increased M/M ratio around the matrix close to osteocytes compared to the bone matrix further away, suggesting localized osteocyte-induced PLR (Gardinier et al., 2016). This finding suggests that PLR regulation takes place predominantly in the mineralized bone matrix such as cortical bone. A better understanding of the mechanism of PLR regulation will aid in developing potential therapeutic applications that could improve cortical bone integrity, which is known to have a lower recovery rate after fracture compared to trabecular fractures (Chen and Sambrook 2011; Rivadeneira and Mäkitie 2016).

Osteocytes are known to secrete signaling factors into the circulatory system to modulate behavior of distant target organs such as parathyroid, kidney, and heart (Martin 2019; Pathak et al., 2020; Florencio-Silva et al., 2015). Particularly, osteocyte-secreted factor, fibroblast growth factor 23 (FGF23) that plays a role in endocrine signaling (Figure 1C). FGF23 contributes to kidney functions, maintaining serum phosphate levels by modulating the expression level of sodium/phosphate co-transporters in the kidney (Bonewald and Wacker 2013; Dallas et al., 2013; Dussold et al., 2019). Through this mechanism, FGF23 suppresses the vitamin D hormone (1,25-dihydroxyvitamin D) production in the kidneys by inhibiting the conversion of 25-hydroxyvitamin D to the active form, 1,25-dihydroxyvitamin D by 1-α-hydroxylase (Martin, David, and Quarles 2012; Dallas et al., 2013). The high FGF23 levels inhibit the vitamin D conversion process leading to decreased phosphate absorption in the intestine. This signaling process is tightly regulated by a feedback system between the active form of vitamin D and the level of FGF23 in circulation, where osteocytes play a key role. The elevated levels of circulating FGF23 are known as a risk factor for heart disease such as left ventricular hypertrophy, but further investigation is required to understand the underlying mechanism of FGF23 in this tissue (Mirza et al., 2009; Desjardins et al., 2012). The high prevalence of heart failure is often seen in chronic kidney disease (CKD) patients, which is also associated with elevated levels of FGF23 (Scialla et al., 2014). Furthermore, the parathyroid gland is another target for FGF23, which decreases PTH secretion. Where increased FGF23 levels modulate the downregulation of PTH mRNA expression and secretion *in vitro* (Krajisnik et al., 2007). The important and well-characterized role of PTH is in maintaining systemic calcium levels, where the parathyroid gland-secreted PTH is known to respond to low serum calcium (Bellido et al., 2013). If there is a high calcium demand in the intestine, the PTH levels increase causing mineral release from bones, which is often seen in pathological conditions such as chronic kidney disease. Osteocytes closely coordinate this process by increasing mineral degradation through PLR. Osteocyte-secreted CatK also contributes to the regulation of PTH levels by increasing parathyroid hormone-related peptide (PTHrP) during lactation (Lotinun et al., 2019).

## Mechanosensors in Osteocytes

Osteocytes are known to be one of the most mechanosensitive cells (Jacobs et al., 2010). These cells can be stimulated by various mechanical forces in bone created by gravitational forces and daily activities leading to changes of interstitial fluid flow and matrix deformation at the cellular level in bone. The osteocyte cellular response to mechanical stimulation is crucial in terms of viability, and also for a regulatory role in balanced bone homeostasis (Qin et al., 2020; Wittkowske et al., 2016). The earlier studies primarily focused on fluid flow-induced osteocyte mechanotransduction compared to direct interaction with extracellular matrix (ECM) deformation (Cheng et al., 2001a; Cherian et al., 2005; Kulkarni et al., 2010; Li et al., 2012; Spatz et al., 2015; Qin et al., 2020). The fluid flow rate used in previous *in vitro* studies was between 0.5 and 2 dynes/cm^2^ (0.5 and 2 Pa) with some studies using up to 16 dynes/cm^2^ (Table 1). These studies were demonstrated using both osteocyte cell lines (Cheng et al., 2001b; Cherian et al., 2005; Kulkarni et al., 2010; Litzenberger et al., 2010; Li et al., 2012; Xu et al., 2012; Spatz et al., 2015; Sato et al., 2020) and primary osteocytes (Ajubi et al., 1996; Klein-Nulend et al., 1997; Sterck et al., 1998; Ajubi et al., 1999; Joldersma et al., 2000). However, the exact physiological flow rate remains unclear. Estimates of the physiological matrix strain that is generated at the cellular level is also lacking. The strain level surrounding osteocytes is heterogeneous, amplifying the strain between the local cellular level and tissue level (Weinbaum et al., 1994; Nicolella et al., 2001; You et al., 2001; Bonivtch et al., 2007; Verbruggen et al., 2012; Hart et al., 2017). Several studies have demonstrated that variations in the size and shape of the LCN geometries are closely associated with non-uniform strain distributions (Canè et al., 1982; Metz et al., 2003). A parametric finite element model used to predict the microstructural response in lacuna showed increased strain with a decreased perilacunar tissue modulus (Bonivtch et al., 2007). The canaliculi diameter was increased by 0.8–1% in response to the applied strain and this deformation directly contributed to the enclosed dendritic process via the tethering elements (e.g., CD44, laminin, and integrins) to the canalicular wall (You et al., 2004). It is postulated that the strain difference between lacunar and canlicular structures may induce significantly different cellular responses in osteocytes (McCreadie and Hollister 1997; Nicolella et al., 2006; Verbruggen et al., 2015). *In vivo* studies revealed that the strain around perilacunar was an order of magnitude greater than the macroscopically applied strains, suggesting that local tissue strain can be magnified by inhomogeneous microstructural features (Nicolella et al., 2006).

**TABLE 1:** Summary table for *in vitro* studies on osteocytes in response to mechanical stimulations. Abbreviations: Sclerostin (*Sost*), cyclooxygenase-1 (*COX-1*), osteoprotegerin (*OPG*), receptor activator of nuclear factor-κB ligand (*RANKL*), podoplanin (*E11*), prostaglandin E2 (PGE_2_), cyclooxygenase-2 (*COX-2*), connexin 43 (Cx43), matrix extracellular phosphoglycoprotein (*Mepe*), phosphate regulating endopeptidase homologue, X-linked (*Phex*), dentin matrix protein 1 (*Dmp1*).

Cell type	Mechanical stimulation	Gene/Protein expression	Outcome
Osteocyte cell lines			
MLO-Y4	Oscillatory fluid flow, 1 Pa/2 h	*COX-2, RANKL/ OPG*	Response of integrin β1 under oscillatory fluid flow. The absence of β1 showed a reduction in *COX-2* and PGE_2_ (Litzenberger et al., 2010)
MLO-Y4	Oscillatory fluid flow, 1 Pa/2 h	*COX-2, Runx-2*, integrin αVβ3, E11	Increased expression of integrin-associated molecules including vinculin, osteopontin, and CD44. Also, more cell spread and fiber stress are formed by fluid flow (Xu et al., 2012; Zhang et al., 2015)
MLO-Y4	Oscillatory fluid flow, 0.5–5 Pa/1–4 Pa	*COX-2, RANKL/ OPG*	Cells were exposed to different shear stress amplitude (0.5–5 Pa), oscillating frequency (0.5–2 Hz), and duration (1–4 h). *COX-2* Upregulated gene expression levels for *COX-2* response to higher shear stress amplitudes, faster oscillating frequencies, and longer flow durations, which direct towards bone formation (Li et al., 2012)
MLO-Y4	Fluid shear stress, 16 Pa/0.5–2 h	*OPG*, Cx43, PGE_2_	Fluid shear stress induces the opening of Cx43 and redistributes Cx43 protein, which promotes PGE_2_ release (Chen and Sambrook, 2001; Cherian et al., 2005)
MLO-Y4	Pulsating fluid flow, 0.7 Pa/1 h	*Mepe, RANKL/OPG*	Pulsatile fluid flow induced *Mepe*, but not *Phex*. *RANKL*/*OPG* gene expression decreased (Kulkarni et al., 2010)
Ocy454	3D fluid shear stress, 0.5–2.0 Pa/2 h or 3 days	*Sost*, *Dmp1*, *RANKL*, *OPG*, *Phex*, *Mepe,* Osteocalcin	Long-term fluid shear stress (3 days) in 2D LS increases *Sost*, *Dmp1*, *RANKL*, *OPG*, *Phex*, *Mepe* (Spatz et al., 2015; Wein et al., 2015)
Ocy454	Laminar fluid flow, 0.8 Pa/45 min	*Sost*	Laminar fluid flow downregulated *Sost* gene expression and demonstrated *HDAC5* is required for loading-induced *Sost* suppression (Sato et al., 2020)
Primary osteocytes			
Chicken osteocytes	Pulsating fluid flow, 0.5 Pa/1 h, 0.7 Pa/10 min	PGE_2_	Osteocytes rapidly respond to fluid flow to increase PEG_2_ (Ajubi et al., 1996)
			Intracellular Ca^2+^ level was increased through mechanosensitive ion channels (Ajubi et al., 1999)
Mouse calvariae	Pulsating fluid flow, 0.7 Pa/1 h	PGHS-2 (Prostaglandin G/H synthase), PGE_2_	After pulsating fluid flow, osteocyte s upregulated *PGHS-2* gene expression, leading to more conversion of arachidonic acid into PGE_2_ (Klein-Nulend et al., 1997)
Human calvarial cells/biopsies	Pulsating fluid flow, 0.7 Pa/1h	PGE_2_, *COX-2*, Nitric oxide	Pulsating fluid flow upregulated PGE_2_, *COX-2*, but not *COX-1* gene expression (Sterck et al., 1998; Klein-Nulend et al., 1998; Joldersma et al., 2000)

**FIGURE 2:**
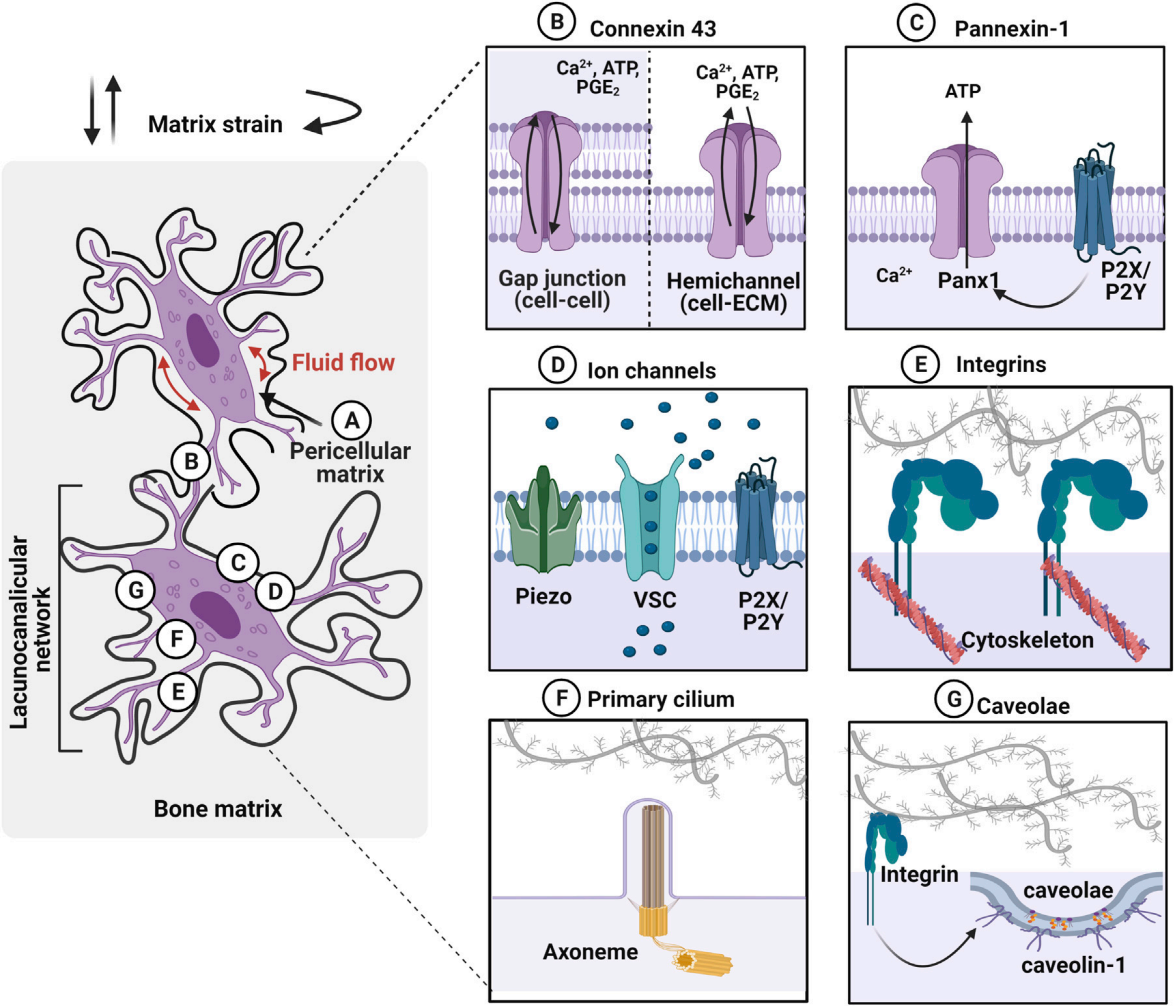
Osteocytes within the lacunocanalicular network express mechanosensors, which can be activated by various mechanical stimuli such as fluid flow in the pericellular matrix and matrix strain (e.g., compressive, tensile, and torsional loading). **(A)** Osteocytes are surrounded by the pericellular matrix, between the cell and the walls of lacunae and canaliculi, which acts as a tether for osteocytes to transduce the mechanical stimulation. **(B)** Gap junctions, expressing on dendritic cell processes, facilitate cell–cell communication between osteocytes. Especially, connexin 43 (Cx43) is highly expressed and these junctions can also function as hemichannels that open to the microenvironment. Mechanical stimuli open these channels and transport calcium ions (Ca^2+^), adenosine triphosphate (ATP), and prostaglandin E2 (PGE_2_) between cells. **(C)** Pannexin-1 (Panx1) hemichannels release ATP to regulate intracellular calcium levels. Panx1 is also associated with purinergic P2X7 receptor to regulate apoptosis. **(D)** Mechanosensing ion channels such as Piezo, voltage-sensitive calcium channel (VSC), and purinergic receptor (P2X/P2Y) are opened in response to the mechanical stimulation and trigger calcium mobilization. **(E)** Integrins, transmembrane receptors that adhere cells to the extracellular matrix through specific motifs, transduce forces into cellular responses by mechanosignaling pathways. **(F)** Primary cilium is a protrusion of the cell membrane that is responsive to stimuli via the ciliary axoneme (microtubules). These immotile membrane protrusions act independently of intracellular Ca^2+^ release. **(G)** Caveolin-1, the structural protein of caveolae is interacting with the integrin β1 subunit to promote mechanotransduction in osteocytes. Figure created using BioRender.

There is a wide variety of potential mechanosensors present on osteocytes, which will be discussed in more detail in the following section, that transduce extracellular signals into cellular responses, including pericellular matrix, connexins/pannexin channels, mechanically-sensitive ion channels, integrins, primary cilium, and caveolae (Figure 2) (Bonewald 2011; Qin et al., 2020). There is an ongoing debate around, whether the osteocyte’s cell body or the dendritic cell processes are the primary mechanosensitive features of osteocytes. The unique dendritic morphology enables osteocytes to create a massively interconnected network in the human body creating a surface area that increases exposure to the surrounding microenvironment (Buenzli and Sims 2015; Hemmatian et al., 2017). A previous study has shown that the dendritic cell processes are more responsive to fluid shear stress than the cell body, using a transwell filter system to separate the dendritic cell processes from the cell body (Burra et al., 2010). Subsequent studies have also concluded that the more sensitive mechanotransduction occurs through dendritic cell processes, which induces calcium influx and regulate gene transcription of key secreted signaling molecules such as sclerostin (Wu et al., 2011; Thi et al., 2013).

### Pericellular Matrix

Osteocytes are surrounded by a layer of the pericellular matrix (PCM) at the interface between the cell membrane and the hard bone matrix (Figure 2A) (Sauren et al., 1992; You et al., 2004). Although the exact composition and structure of PCM are not well defined around the osteocytes, it is considered to be comprised of collagen, fibronectin, proteoglycans, glycoproteins, hyaluronic acid and perlecan/HSPG2 (Sauren et al., 1992; You et al., 2004; Weinbaum et al., 2007; Thompson et al., 2011a; Burra et al., 2011). It was observed that the transverse fibers span the entire PCM, which facilitate the direct interaction of osteocyte dendritic process to the canalicular wall with possible tethering molecules such as integrins, laminin, and CD44 (Noonan et al., 1996; You et al., 2004). It has been proposed that fluid drag forces transduced on the PCM via tethering molecules may induce osteocyte mechanotransduction by amplifying the strain at the cell membrane (You et al., 2001; Han et al., 2004). The strain amplification was further investigated in the context of integrin attachment points along the osteocyte dendritic processes with the collagen hillock traversing the PCM (Wang et al., 2007). This study demonstrated that the direct interaction of integrin promoted strain amplification by more than two orders of magnitude compared to the tissue-level strain. Hyaluronic acid has been suggested as a major component of the PCM surrounding the osteocytes (Nakamura et al., 1995; Noonan et al., 1996). This was confirmed by diminished osteocyte PGE_2_ release with a hyaluronidase treatment after being exposed to oscillating fluid flow under *in vitro* conditions (Reilly et al., 2003). The disappearance of integrin α5 was also observed with hyaluronidase treatment suggesting a tethering element of integrin is closely associated with the hyaluronic acid of PCM (Burra et al., 2011). A reduced volume of hyaluronic acid in PCM was observed with aging, which is possibly associated with the change in mechanoresponse of the osteocytes (Wang et al., 2014; Hagan et al., 2020). Perlecan, a large proteoglycan is also known to regulate solute transport and mechanosensing in PCM (Thompson et al., 2011b). Mice with perlecan deficiency showed decreased anabolic stimuli compared to the control group suggesting osteocytes experienced less fluid drag force, an effect also seen in aged mice (Wang et al., 2014).

### Connexin/Pannexin Channels

Connexins are pore structure in the plasma membrane of osteocytes forming either gap junctions (cell–cell) or hemichannels (HC) (cell–matrix) (Figure 2B) (Plotkin and Bellido 2013). Although connexin 43 (Cx43) is the most highly expressed connexin in all the bone cell types, Cx37 has also been detected in osteocytes (Jones et al., 1993). This enables osteocytes to communicate with each other by the transfer of small molecules (less than 1 kD) through these gap junctions and respond to the environment via hemichannels that open to the extracellular space. Once osteocytes receive mechanical stimulation, Cx43 is phosphorylated, inducing the opening of connexons, six connexin subunits forming intercellular channels to regulate several effects such as influx of Ca^2+^, ATP, and PGE_2_ from the extracellular environment (Riquelme et al., 2021; Cherian et al., 2005; Genetos et al., 2007; Cheng et al., 2001a; Riquelme and Jiang 2013). This mechanism promotes the extracellular signal-regulated kinase (ERK)1/2-mitogen-activated protein kinase (MAPK) pathway, which regulates the bone remodeling process and is known to inhibit osteocyte apoptosis (Plotkin et al., 2005). Conversely, prolonged closure of connexins due to reduced mechanical loading or aging activates protein kinase B (Akt)/P27/Caspase-3 pathway leading to apoptosis. Pannexin-1 (Panx1) is another mechanosensitive channel expressed in osteocytes that forms only non-junctional channels to exchange small molecules between cell–extracellular space in response to mechanical stimulation (Figure 2C) (Aguilar-Perez et al., 2019). During apoptosis, Panx1 channel can be activated by coupling with the purinergic receptor, P2X7 to release ATP to send signals for macrophages (Sandilos et al., 2012). Panx1 knockout mouse model demonstrated that load-induced periosteal bone formation was diminished by dysregulated β-catenin and sclerostin expression in osteocytes (Seref-Ferlengez et al., 2019).

### Mechanically-Sensitive Ion Channels

Mechanically-sensitive ion channels (MSICs) in osteocytes are responsive to mechanical stimulation, by opening in response to the tension created in the plasma membrane (Figure 2D) (Li et al., 2019a). The role of the mechanosensing ion channel, Piezo 1, which facilitates the exchange of ions between cell and extracellular environment, and leads to the opening of voltage-sensitive calcium channels (VSCs). Osteocytes primarily express more T-type CaV3.2 VSC subunits and a relatively small amount of L-type α1 subunits, which accelerate ATP/Ca^2+^ release in response to fluid shear stress (0.5–4 Pa) (Thompson et al., 2011a; Lu et al., 2012). Piezo 1 has been shown to not only modulate intracellular calcium levels, but also activate downstream signaling pathways such as Akt-sclerostin in response to cyclic stretch-induced mechanical stimulation. Here, sclerostin expression was downregulated by Akt phosphorylation, which was confirmed by the Piezo1 knock-out, which resulted in diminished calcium influx and Wnt, and release of ATP from the cell (Robling and Turner 2009). Osteocytes furthermore regulate mechanically induced ATP *via* P2X/P2Y receptors leading to purinergic signaling (Li et al., 2005; Burnstock et al., 2013).

### Integrins

Integrins are heterodimeric transmembrane cell receptors composed of alpha (*α*) and beta (*β*) subunits that anchor cells through specific matrix motifs transducing mechanical dynamics from matrix strain and fluid-flow shear stress (Figure 2E) (Geoghegan et al., 2019). Osteocytes are known to differentially express integrins, with the α5β1 integrins localizing strongly on the cell body, and αVβ3 integrins along the dendritic cell processes, suggesting site-directed osteocyte mechanotransduction (Haugh et al., 2015; Geoghegan et al., 2019). In extracted mouse bone tissue, integrin αVβ3 binding was observed to localize to the canalicular wall along the periodic protrusions (McNamara et al., 2009). It is proposed that proteoglycan tethering elements bridging the dendritic process of osteocytes to the canalicular wall *via* integrin αVβ3 promotes interaction with the ECM proteins containing Arginine-Glycine-Aspartic acid (RGD) sequence motifs such as fibronectin, osteopontin, von Willebrand factor, sialoprotein, and thrombospondins, but not to collagen (Haugh et al., 2015). The direct adhesion between osteocyte and ECM facilitates the formation of focal adhesions, which link to the actin skeleton to activate cellular responses, such as regulating secreted signaling molecules that are guiding the effector cells. Integrins are known to recruit focal adhesion proteins, including vinculin and paxillin, which link the cytoskeleton to the ECM. Both *in vivo* and *in vitro* studies demonstrated the expression of focal adhesion proteins, such as vinculin, in osteocytes (Zhou et al., 2019; Cao et al., 2020). Another study suggested that integrin αVβ3-mediated mechanotransduction lacks the classic focal adhesion protein recruitment, but rather mediates Ca^2+^ signaling, ATP release and membrane potential through the purinergic channel pannexin 1, the calcium channel CaV3.2-1, and the ATP-gated purinergic receptor P2X7 (Cabahug-Zuckerman et al., 2018). Furthermore, both α5β1 and αVβ3 integrins are known to activate Ca^2+^ channels, but through different mechanisms. An earlier study has identified integrin αVβ3-specific intracellular Ca^2+^ signals, using a novel technique called Stokesian fluid stimulus probe (SFSP). This probe enables the application of hydrodynamic forces (pN range) to the discrete location of the cell body and dendritic cell processes (Thi et al., 2013). The SFSP-stimulated osteocytes (MLO-Y4) showed that dendritic cell processes were more mechanosensitive in the piconewton range of mechanical stimulation, resulting in increased levels of intracellular Ca^2+^. Using an integrin αVβ3- specific antagonist, Integrisense 750, diminished Ca^2+^ response under SFSP-stimulation was observed (Thi et al., 2013). Thus, integrin αVβ3 is not only involved in activation of focal adhesion protein-mediated mechanotransduction, but also regulates intracellular Ca^2+^ signals through cation and stretch-activated channels in osteocytes. Interestingly, the α5β1 integrins are directly associated with the opening of Cx43 HC to release anabolic molecules from osteocytes (PGE_2_), in response to fluid shear stress (Batra et al., 2012). PGE_2_ also has an autocrine effect, stimulating the upregulation of Cx43 protein expression in osteocytes, which further induces an increase in formation of gap junctions between cells (Cheng et al., 2001b). The activation of the intracellular mechanotransduction pathway, involving phosphoinositide 3-kinase (PI3K)-Akt signaling to open Cx43 HC by conformational activation of integrin α5β1 is independent of adhesion to the ECM. Especially, the integrin α5 subunit is crucial in establishing the specific interaction with the C termini of Cx43. It was observed that siRNA knockdown of integrin α5 diminished the opening of the Cx43 HC under fluid flow-induced stimulation (Batra et al., 2012; Riquelme et al., 2021). It was argued that not only integrin α5 activates Cx43 HC, but also integrin αVβ3 expressed along the dendritic cell processes can transduce signals to the cell body for Cx43 HC activation *via* PI3K-Akt signaling. This was demonstrated both *in vitro* and *in vivo* under fluid shear stress with steady fluid flow/oscillatory fluid flow and under tibial compression in mice. The results showed that integrin αV was more responsive to low fluid shear stress levels to activate Cx43 HC compared to integrin α5 induced activation. Notably, at a higher fluid shear stress level, integrin α5 was activated independently of integrin αV, implying that the activation of either integrin pair is fluid shear stress level dependent. This study concluded that fluid shear stress could not suppress sclerostin expression without Cx43 HC, which was demonstrated by blocking with antibodies, suggesting Cx43 is essential for the anabolic effects on bone.

Numerous *in vitro* studies have been undertaken to understand targeted integrin-mediated mechanotransduction in osteocytes, with only a few *in vivo* studies, with most of these using specific integrin β1-deleted transgenic mice (Zimmerman et al., 2000; Litzenberger et al., 2009; Shekaran et al., 2014). In a study investigating the integrin β1-mediated response after cyclic ulna loading for 3 days, osteocyte-specific integrin β1-knockout mice showed reduced bone formation suggesting that the integrin β1 is required to promote mechanically-induced bone formation (Litzenberger et al., 2009). Unfortunately, the osteocyte-specific integrin β3 targeted approach has not been progressed due to technical challenges. For this reason, it is still not clear what the precise functional roles that these integrins play on bone homeostasis are.

### Primary Cilium

Cilia are present in both motile and immotile cells, which have microtubule axoneme. Nine sets of microtubules doublets provide structural support and rigidity (Satir et al., 2010). The primary cilium has “9 + 0” pattern with nine doublet microtubules without the central pair, which are seen in the immotile cilia. In contrast, “9 + 2” pattern with 9 doublets plus one central pair of microtubules is often seen in motile cilium. Osteocytes present non-motile primary cilium with “9 + 0” arrangement, 2–9 µm in length, which are mechanoresponsive (Figure 2F) (Qin et al., 2020). Primary cilium changes the morphology during mechanical adaptation, which induces expression of cilium-related proteins such as Sperm flagellar protein 2 (Spef2), polycystin -1 or -2 (PC1 or 2), kinesin II intraflagellar transport (Kif3a), and Adenylyl cyclase 6 (AC6) (Xiao et al., 2006; Temiyasathit et al., 2012; Qin et al., 2020). Primary cilium also changes its stiffness in response to mechanical stimulation through an acetylation-mediated mechanism that induces calcium movement. This mechanism is dependent on polycystines (polycystin 1 and 2). These are proteins located at the base of the cilium acting like a cationic change to facilitate Ca^2+^ transfer (Yavropoulou and Yovos 2016). Interestingly, polycystin 1 mutant mice showed reduced bone mineral density due to a lack of response to mechanical stimulation (Xiao et al., 2006). Mice also showed decreased OPG and increased RANKL levels that results in reduced bone mineral density in both trabecular and cortical bones (Temiyasathit and Jacobs 2010). The gene expression level of runt-related transcription factor 2 (*Runx2*), osterix, and osteocalcin were also observed to decrease, where these are all key parameters responsible for bone development, bone density, and mechanical properties.

### Caveolae

Although this has been demonstrated to date only in MLO-Y4 osteocytic cells (Figure 2G), caveolin-1, the structural protein of caveolae was proposed as a membrane mechanosensor in osteocytes, interacting with the integrin β1 subunit (Gortazar et al., 2013). Caveolae are 60–80 nm plasma membrane pits that are present in many mechanosensitive cells such as myocytes. In osteocytes, caveolae are physically linked to integrin β1 leading to activation of ERK through tyrosine protein kinase (Src) and focal adhesion kinase (FAK) phosphorylation. Thus, it was postulated that caveolin-1 is essential for integrin/Src/ERK activation of pro-osteocyte survival mechanisms. This was confirmed by inhibition of caveolin-1 that diminishes anti-apoptotic effects of mechanical stimulation due to disrupted ERK activation (Plotkin et al., 2005). The detailed underlying mechanisms around the role of caveolin-1 in mechanosensing in osteocytes are unclear, however, this integrin-dependent mechanism is intriguing.

## Mechanotransduction Pathways in Osteocytes and Therapeutic Implications

Although mechanically-induced osteocyte responses have been studied extensively, the precise signaling pathways underlying these responses are still unclear. Understanding the signaling pathways is critical due to the implications of the functional outcomes for both bone health and diseases, as well as more broadly for the other systemic role of osteocytes through their endocrine functions (Dallas et al., 2013). For the past decades, several signaling pathways have been identified, as potential therapeutic targets to improve bone health (Table 2).

**TABLE 2:** The key research demonstrations for mechanosensitive signalling pathways in osteocytes and therapeutic implications.

Signalling pathway	Research	Clinical implications	Reference
Sphinogolipid	SP1 induces osteoclast precursor migration thus increase bone resorption	Increased S1P for osteoporotic fracture/low bone mineral density	(Tian et al., 2021; Thuy et al., 2014; Zhang et al., 2015)
Wnt/β-cat	β-catenin is required for osteocyte viability	Bisphosphonates, prostaglandin, estrogen are known to prevent osteocyte apoptosis	(Bellido 2014;Xia et al., 2010; Kamel et al., 2010; Plotkin et al., 1999; Kitase et al., 2010; Tomkinson et al., 1998; Duan and Bonewald 2016; Lin et al., 2020)
	β-catenin is associate with FoxO transcription to prevent osteocyte apoptosis		
	β-catenin binds to the connexin 43 promoters, promoting cell-cell interaction and enhance the viability		
AMPK	AMPK is the regulator for cellular energy homeostasis	Osteoporosis is possibly a disorder of energy metabolism	(Tong, Ganta, and Liu 2020; Jeyabalan et al., 2012; Ru and Wang, 2020)
	AMPK increases cellular AMP/ATP ratio helps to maintain energy homeostasis	AMPK can be activated by antidiabetic drugs (metformin and thiazolidinediones)	
	Protect osteocyte apoptosis by suppressing oxidative stress		
FoxO	FoxO activation inhibits osteocyte apoptosis induced by aging and unloading	Targeting aging-related osteoporosis/bone fragility fractures	(Kawata and Mikuni-Takagaki 1998; Ru and Wang 2020; Domazetovic et al., 2017)
	FoxO signalling associate with Wnt/β-cat for osteocyte viability	ROS induce apoptosis; antioxidants such as polyphenols and anthocyanins through diet intake induce anti-osteoclastogenic action	
PTH	Activation of PTH receptor suppressed sclerostin expression	Homologous with PTH (N-terminal 1–36) and PTH-related protein (C-terminal 107–109) induce bone formation and also reduce oxidative stress	(Kamel et al., 2010; Collette et al., 2012; Wysolmerski 2012; Bellido et al., 2013; Maycas et al., 2015; Portal-Núñez et al., 2016)
	Increased level of PTHrP activate PTH receptor for anti-apoptotic effect		
	Deletion of *Mef2C* in osteocytes induced bone formation by decreasing sclerostin;		
	PTH activates Wnt receptor, LRP6 directly, or through FoxO degradation to stabilise beta-catenin in Wnt signalling to induce osteogenesis	Antioxidant supplement (Resveratrol)	

### Sphingolipid Signaling Pathway

**FIGURE 3:**
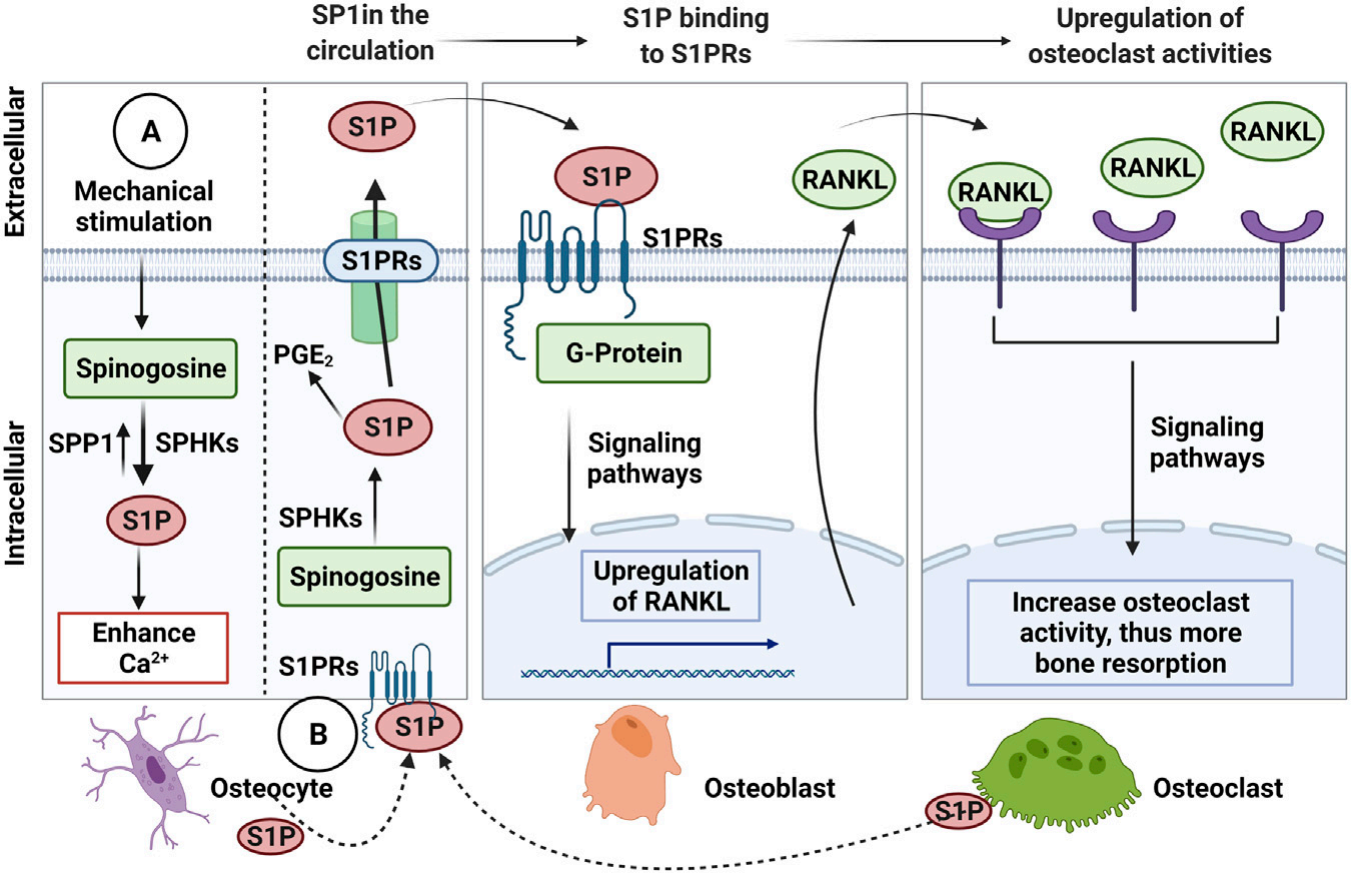
The Sphingosine-1-Phosphate (S1P) signalling in osteocytic mechanotransduction and effects of osteocyte-mediated extracellular S1P on osteoblast-osteoclast crosstalk. **(A)** The endogenous S1P production in response to mechanical stimulation from Sphinogosine by the S1P phosphohydrolase (SPP1) and sphinogosine kinase (SPHKS) leading to increased cellular Ca^2+^. **(B)** S1P can be released by osteocytes, which extracellular S1P can bind to S1P receptors (S1PRs) on osteoblasts that activate signaling pathways to upregulate receptor activator NF-κB (RANKL). Then, osteoblasts release RANKL that binds to RANK on osteoclasts to increase osteoclast activity for bone resorption. Osteoclasts are also known to release S1P, which binds to S1PRs on osteocytes as a feedback loop to increase intracellular S1P and prostaglandin E2 (PGE_2_), Receptor activator NF-κB (RANKL), ligand (RANKL). Figure created using BioRender.

**FIGURE 4:**
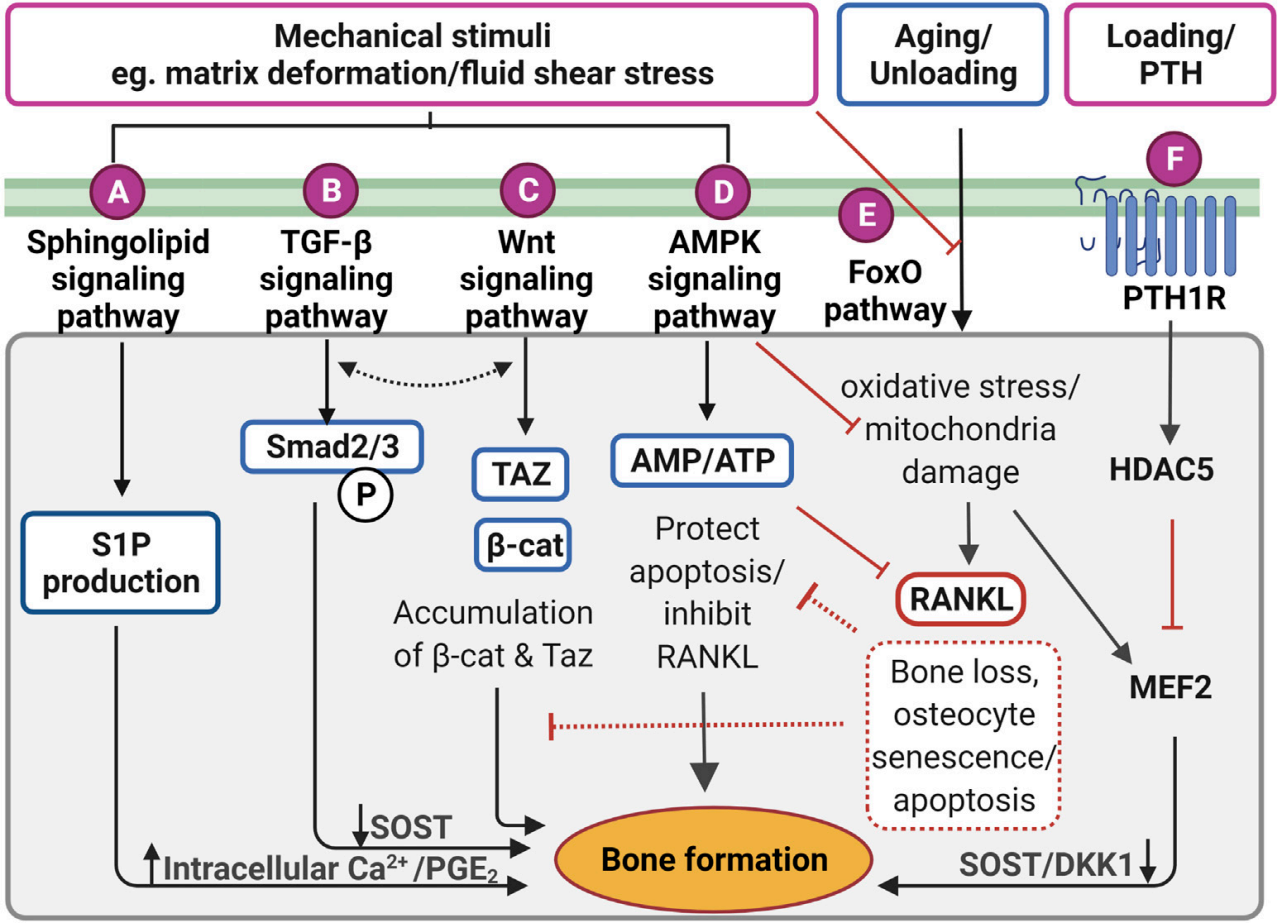
Proposed mechanotransduction pathways in osteocytes for therapeutic targets showing intracellular signaling in response to the mechanical stimulation. **(A)** Pulsatile fluid flow triggered sphingolipid signaling to regulate the lipid mediators such as sphingosine-1-phosphate (S1P) production that upregulates the intracellular calcium ions (Ca^2+^) levels and prostaglandin E2 (PGE_2_) synthesis/release in osteocytes. **(B)** Fluid shear stress upregulates suppressor of mothers against decapentaplegic 2/3 (Smad2/3) phosphorylation triggering transforming growth factor-beta (TGF-β) signaling, resulting in sclerostin (SOST) downregulation. This is independent of TGF-β receptor-induced response. **(C)** Wnt/β-catenin signaling can be elicited by direct response to extracellular matrix deformation via integrins or fluid shear stress, which is important to maintain osteocyte viability and anabolic effect by accumulating Taz and β-catenin (β-cat). Interestingly, both TGF-β and Wnt/β signaling may interact with each other to induce bone formation, however, the exact mechanism is not clear. **(D)** Under mechanical stimuli, adenosine monophosphate (AMP)-activated protein kinase (AMPK) signaling governs energy homeostasis in osteocytes by increasing the AMP/adenosine triphosphate (ATP) ratio for inhibiting apoptosis and decrease receptor activator of nuclear factor-κB ligand (RANKL) expression. **(E)** Forkhead box O (FoxO) signaling is activated to protect osteocytes from oxidative stress and mitochondria damage caused by aging and reduced mechanical stimulation. Without FoxO activation, osteocytes lead to senescence and apoptosis. **(F)** Parathyroid hormone receptor (PTHr) is activated both by mechanical stimulation as well as parathyroid hormone. This receptor upregulates histone deacetylase 5 (HDAC5), which inhibits myocyte enhancer factor 2 (MEF2C), responsible for negative Wnt signaling molecules, SOST and dickkopf-related protein 1 (DKK1). Figure created using BioRender.

In osteocyte cell models (MLO-Y4 and Ocy454 cell line), intracellular sphingosine-1-phosphatase (S1P) levels were found to be upregulated in response to fluid flow mechanical stimulation, with a corresponding downregulation of the enzymes for degradation/dephosphorylation of S1P (Sgp11, Sgpp11), as well as upregulation of Sphk1, responsible for phosphorylation of S1P leading to its activation (Figure 3A) (Zhang et al., 2015; Dobrosak and Gooi 2017). In response to mechanical load, S1P in osteocytes acts as a signaling molecule for modifying cellular Ca^2+^ levels and PGE_2_, either directly via intracellular S1P or indirectly via S1P binding to G-protein-coupled receptors (Zhang et al., 2015; Meshcheryakova et al., 2017). In response to mechanical stimulations, osteocytes modulate S1P production and secretion that facilitate paracrine osteoblast-osteoclast crosstalk. In general, osteocyte-secreted S1P plays important role in both osteoblast and osteoclast activities (Figure 3B) (Zhang et al., 2020). The newly synthesized S1P is released intracellularly and acts like a second messenger, which induces Ca^2+^ release in an IP3-independent manner. The extracellular S1P can also bind to G-protein-coupled receptors (S1P receptors, SIPRs), which increases the mobilization of the intracellular level of Ca^2+^. Furthermore, intracellular S1P can be released into circulation and binds to S1PRs on osteoblasts promoting cell differentiation and also inducing *RANKL* expression (Dobrosak and Gooi 2017). The osteoblast cells produce RANKL and this binds to the receptor RANK to activate osteoclasts, suggesting the crosstalk between osteoblasts-osteoclasts is important to mediate the balance between bone formation and resorption. The loop of this crosstalk is regulated by osteocytes since S1P secreted by osteoclasts is released and binds to S1PRs on osteocytes in a feedback loop mechanism. The sphingolipid signaling pathway is activated in response to oscillatory fluid flow-induced loading in bone (Figure 4A) (Tian et al., 2021; Thuy et al., 2014). The lipid mediator, S1P is the sphingolipid metabolite that acts as a signaling molecule for modifying intracellular Ca^2+^, which was shown in both osteoblasts and osteoclasts previously (Meshcheryakova et al., 2017).

Interestingly, the increased S1P level in blood (>200 nM) is closely associated with bone fracture risk and low bone mineral density (Lee et al., 2012). The blood S1P plasma levels have been observed to be elevated in postmenopausal women compared to premenopausal women, with the postmenopausal women known to be at higher risk of bone loss (Ardawi et al., 2018). In pathological conditions, the S1P disrupts the equilibrium between osteoblast and osteoclast activities. Increased production of S1P by osteocytes in response to mechanical stimulation may also promote the osteoblast differentiation process, which results in a decreased level of osteoblast-produced RANKL inhibiting osteoclast differentiation (Dobrosak and Gooi 2017). There are S1P-targeted therapeutic approaches for osteoporosis using S1P lyase inhibitors (e.g., CYM5520 and LX2931) and a structural analog of sphingosine (e.g., FTY720, fingolimod) (Tian et al., 2021). These pharmacological treatments increase S1P at tissue levels, inducing new bone formation, which was confirmed in ovariectomized mice and rat studies (Huang et al., 2016; Weske et al., 2019). Currently, however, it remains unclear whether the expression of S1P receptors in osteocytes has a key regulatory role in response to S1P in the blood, and therefore further studies are required.

### TGF-β Signaling Pathway

TGF-β signaling is also responsive to mechanical stimulation independent from TGF-β receptor-induced responses, which are initiated by Smad2/3 phosphorylation and downregulates sclerostin (Figure 4B) (Nguyen et al., 2013). The level of Smad2/3 phosphorylation was elevated even in the presence of the TGF-β receptor inhibitor, confirming fluid shear stress directly triggered TGF-β signaling (Monteiro et al., 2021). Also, the level of Smad2/3 phosphorylation was larger under fluid shear stress compared to osteocytes with TGF-β treatment, suggesting TGF-β signaling is largely induced by fluid shear stress. Impaired TGF-β signaling is often associated with aging, diminished mechanical adaptation and low bone mass. A recent *in vivo* study revealed that TGF-β signaling is important for osteocyte functions in LCN such as PLR, as mentioned previously (Schurman et al., 2021). Deletion of this specific TGF-β signaling compromised osteocytes functional response to mechanical stimulation, similar to that observed with aging.

### Wnt/β-Catenin Signaling Pathway

The Wnt/β-catenin signaling has a crucial role in bone formation, not only for the effector cells but also in self-regulatory mechanisms for osteocytes (Figure 4C). Osteocytes increase the expression of the Wnt ligand in response to mechanical stimulation. Osteocyte-produced Wnt can then bind to the LRP6 receptor on osteocytes leading to intracellular β-catenin accumulation in the cytoplasm altering gene transcription changes for Wnt antagonists, *SOST*, and *DKK1* (Bonewald and Johnson 2008; Tu et al., 2015). In response to mechanical loading, the Wnt/β-catenin signaling pathway plays an important role, not only for bone anabolic effects but also in osteocyte viability (Bonewald and Johnson 2008). Glucocorticoid treatment (dexamethasone) can cause secondary osteoporosis by inducing apoptosis in osteocytes and interestingly, this glucocorticoid-induced apoptosis can be inhibited by a steady laminar fluid shear stress of 1.6 Pa for 2 h (Kitase et al., 2010). The protective mechanism is mediated through the release of an osteocyte-produced signaling molecule called PGE_2_ associated with Wnt/β-catenin signaling by pulsatile fluid flow shear stress (0.2–2.4 Pa for 1 h), which is independent of LRP5 receptors in osteocytes (Kamel et al., 2010). This protective effect was induced through PGE_2_ binding to EP2/4 receptors, which leads to Akt activation for glycogen synthesis kinase 3 (GSK-3β) inhibition. This results in an accumulation of intracellular β-catenin in osteocytes. Through this process, PGE_2_ can also induce anabolic bone formation by crosstalk with Wnt/β-catenin pathway leading to downregulation of *SOST* and *DKK1* transcription levels and increased expression of *Wnt* in osteocytes. β-catenin is also known to bind to the Cx43 promoters, upregulating *Cx43* transcription. This enhances osteocyte cell–cell communication for osteocyte viability and increases PGE_2_ levels in response to steady laminar flow of 1.6 Pa for 2 h (Cherian et al., 2003; Xia et al., 2010). This mechanism is also important to the integrin β1-caveolin-1 induced signaling, where vascular endothelial growth factor receptor 2 (VEGFR2) associated with caveolin-1 was reported to be responsive to 1 Pa fluid flow shear stress after 10 min, inducing Wnt/β-catenin signaling (de Castro et al., 2015). Another study also observed that VEGFR2 was activated by pulsatile fluid flow shear stress (1 Pa for 10 min) *via* caveolin, which induces ERK phosphorylation leading to β-catenin translocation to the cell membrane and triggering osteocyte prosurvival signaling. The deletion of caveolin-1 by siRNA impaired VEGFR2 activation, inducing osteocyte apoptosis (Gortazar et al., 2013).

It is known that the Wnt/β-catenin signaling pathway is in crosstalk with various other signaling pathways in response to mechanical stimulations. The TGF-β signaling pathway is known to interact with Wnt/β-catenin signaling in response to mechanical stimulation (Guo and Wang 2009; Rys et al., 2016). Although the mechanism behind the association is still not fully understood, these pathways are associating at multiple hierarchical levels to regulate common target genes, such as sclerostin. This mechanism is associated with the Forkhead box O (FoxO) signaling pathway to inhibit osteocyte apoptosis, which will be further explained later (Manolagas and Almeida 2007). From a therapeutic perspective, Wnt/β-catenin signaling can be induced by bisphosphonates, prostaglandin, estrogen, and anti-sclerostin antibodies, which are all known to prevent osteocyte apoptosis and have anabolic bone effects (Tomkinson et al., 1998; Plotkin et al., 1999; Kitase et al., 2010).

### AMPK Signaling Pathway

RNA-sequencing analysis showed significantly up-regulated 5′adenosine monophosphate-activated protein kinase (AMPK) signaling pathways in osteocytes under fluid shear stress (Figure 4D) (Govey et al., 2015). Prior to this study, the same group also demonstrated the rapid release of ATP in response to fluid shear stress together with up-regulation of the ATP-producing enzyme, nucleoside diphosphate kinase B (NDK), suggesting initiation of AMPK signaling to generate more ATP (Govey et al., 2014). Osteocytes have also been shown to activate AMPK signaling pathway under energy imbalance conditions, like high oxidative stress or nutrient suppression as a protective mechanism (Tong et al., 2020). AMPK is a heterotrimeric complex including α, β, and γ subunits. This signaling pathway can be triggered by phosphorylation of AMPK via catalytic α subunit in low energy status, which can be detected *via* the increased ratio of adenosine monophosphate (AMP)/ATP, by turning on ATP-producing catabolic pathways and turning off ATP-consuming anabolic pathways to restore energy (Jeyabalan et al., 2012). This process is often found in autophagy, which is a survival mechanism to prevent osteocyte apoptosis that can be found under reduced mechanical stimulation. Interestingly, it was shown that AMPK activity is associated with bone metabolism by using a 5-Aminoimidazole-4-carboxyamide ribonucleotide (AICAR), an analog of AMP for AMPK activation (Yokomoto-Umakoshi et al., 2016). Osteocytes (MLO-Y4 cell line) with AICAR treatment induced the phosphorylation of AMPK α subunit leading to significantly reduced *RANKL* gene expression, suggesting inhibition of osteoclast activity (Yokomoto-Umakoshi et al., 2016; Tong et al., 2020). Interestingly, AMPK activation in osteocytes was found to regulate *FGF23* transcription in response to mineral metabolism (Komaba 2018). For example, deficient Ca^2+^ stores in the endoplasmic reticulum of osteocytes stimulate store-operated calcium entry (SOCE) *via* Orai1 on the cell membrane, which induces the influx of Ca^2+^ from the extracellular microenvironment leading to *FGF23* transcription. Conversely, in CKD, AMPK is activated due to decreased levels of ATP, which blocks the Ca^2+^ influx leading to inhibition of *FGF23* transcription in osteocytes leading to imbalanced serum calcium and phosphate levels. This mechanism, which still needs further investigation, may represent an important therapeutic target for CKD patients.

### Fox Signaling Pathway

The protective (anti-apoptotic) action of the FoxO signaling pathway in osteocytes, as has been observed for other cell types, is also activated by mechanical stimulation (Figure 4E) (Ambrogini et al., 2010). Long-lived osteocytes experience oxidative stress and mitochondrial damage leading to apoptosis under physiological conditions such as reduced level of mechanical stimulation and aging. For example, increasing oxidative stress due to aging leads to bone loss, which is closely associated with reactive oxygen species (ROS), inhibiting the translocation of FoxO into the nucleus. However, in response to mechanical stimulation, FoxO is phosphorylated *via* PI3K/Akt signaling pathway, which increases β-catenin associated with the FoxO transcription factor. Osteocyte viability is important for balanced bone homeostasis as osteocyte apoptosis often leads to disease states and upregulation of bone resorption. Osteocyte apoptosis upregulates the expression of sclerostin and RANKL, promoting increased osteoclast activities (Zhang et al., 2019a). Interestingly, osteocytes located in deeper cortical bone showed abundant mitochondria with high levels of glycolytic enzymes, suggesting more protection against oxidative stress (Frikha-Benayed et al., 2016). Effective FoxO activation is closely associated with the Wnt/β-catenin signaling pathway, which is also responsible for osteocyte viability (Zhang et al., 2019b). An earlier study in FoxO-deficient mice showed increased osteocyte apoptosis leading to decreased osteoblast activities, resulting in reduced bone mass in these animals (Ambrogini et al., 2010). FoxO signaling pathway represents a potential target during aging and the observed decrease in osteocyte number that occurs, potentially through the use of antioxidant supplements such as polyphenols, anthocyanins to inhibit osteocyte apoptosis (Domazetovic et al., 2017; Ru and Wang 2020).

Osteocyte-secreted sclerostin expression was once thought to be regulated only by PTH, however, the recent findings demonstrate it is also induced by the fluid flow shear stress on osteocytes (Figure 4F) (Spatz et al., 2015; Sun et al., 2019; Sato et al., 2020). Upon stimulation, histone deacetylase 5 (HDAC5) inhibits myocyte enhancer factor 2 (MEF2C), responsible for *SOST* transcription in osteocytes. As expected, overexpression of HDAC5 in osteocyte cells downregulated *SOST* expression (Baertschi et al., 2014; Wein et al., 2015). Conversely, *HDAC5* knockout mice showed an upregulation of sclerostin mRNA levels, and of the number of sclerostin-positive cells leading to a diminished Wnt/β-catenin signaling pathway in osteoblasts (Wein et al., 2015).

## Osteocyte-Related Diseases and Treatments

Abnormalities of bone strength and microstructure are common in bone diseases, where the bones become more fragile and are more likely to fracture (Feng and McDonald 2011). These disorders are often closely associated with the dysregulation of bone cells, especially osteocytes (Pathak et al., 2020). Specifically, the loss of osteocyte functional ability is linked to compromised bone homeostasis. Osteocyte apoptosis has been proposed as a major risk factor caused by aging, reduced physical activity, hormone deficiency and inflammation resulting in dramatic decrease of osteocyte density (Almeida 2012). As a result, aged or dying cells are no longer able to carry out functional roles, which have an impact on the bone matrix quality (Shah et al., 2018). The accumulation of apoptotic osteocytes with aging is linked to several bone diseases such as osteonecrosis and the onset of age-related osteoporosis leading to increased fracture risk. During apoptosis, osteocytes secrete signals to osteoclasts to be recruited to the site for bone resorption (Schaffler et al., 2014). When apoptosis takes place, osteocytes release damage-associated molecular patterns (DAMPs) for osteoclast recruitment (McCutcheon et al., 2020). When dead osteocytes are removed, the empty lacunae are hyper-mineralized with calcium phosphate, leading to a condition called micropetrosis, resulting in more brittle bones (Qiu et al., 2002; Bell et al., 2008). The accumulation of mineralization in lacunae also interrupts the osteocytic cell–cell communication, leading to depletion of signals and nutrition due to disturbance of canalicular fluid flow (Hemmatian et al., 2017). This cascading process then inevitably further affects other osteocytes resulting in even more extensive osteocyte apoptosis. With less active osteocytes, the bone is less likely to be protected against microdamage. Microdamage triggers dying osteocytes to send signals for osteoclast activation, and at the same time, osteocytes also send anti-apoptotic factor, BAX to neighboring cells to protect their viability (Verborgt et al., 2000; Bonewald 2017). By doing this, the number of apoptotic osteocytes can be minimized around the damaged area. However, if there is a decreased number of viable cells, this mechanism is disrupted, leading to a large area of microcracks (Ma et al., 2008). Therefore, osteocyte cell viability plays a crucial role in the maintenance of bone health, and also protects against microdamage, which is a normal physiological process. The apoptosis process is closely associated with increased oxidative stress, which was confirmed with oxidative stress markers such as p53 and p66^Shc^ in aged mice (Almeida et al., 2007). The oxidation process has been shown to be delayed by anti-oxidant *N*-acetyl Cysteine. Another noticeable change in aged osteocytes is the decreased level of autophagic activity, which is an important indicator for stress susceptibility (Ru and Wang 2020). For example, aged-osteocytes are less likely to produce autophagic proteins (e.g., Beclin-1) to suppress apoptotic proteins (e.g., cleaved-caspase-3). This mechanism is important especially for the anti-apoptotic activity of neighboring cells. However, prolonged stress will cause apoptosis eventually, which highlights the importance of the underlying mechanism between autophagy and apoptosis. Apart from aging, other factors such as estrogen deficiency and glucocorticoid treatment can also induce osteocyte apoptosis leading to osteoporosis (Jilka et al., 2013). Additionally, inflammatory cytokines such as interleukin 1 (IL-1) and tumor necrosis factor-alpha (TNF-α) increase osteocyte death (Marahleh et al., 2019; Wang et al., 2019). Some factors including parathyroid hormone, estrogen, bisphosphonates are known to protect osteocytes from apoptosis (Plotkin et al., 1999; Bellido and Plotkin 2011). Furthermore, as already mentioned above, unloading or decreased level of exercise often leads to decreased bone mass, which is well described in astronauts or bedridden patients (Bradbury et al., 2020; Stavnichuk et al., 2020). These findings are further illustrating, that osteocytes require mechanical stimulation, which can be introduced by mechanotherapy such as low-intensity pulsed ultrasound (LIPUS) treatments where this type of direct mechanical stimulation has been shown to improve bone healing (Thompson et al., 2016; Jiang et al., 2019). The vibration therapy studies demonstrated that high-frequency, low-magnitude vibration therapy (gravitational force = acceleration of 9.81 m/s^2^, frequency >30 Hz) improved bone health (Thompson et al., 2014). These relative parameters were estimated based on the bone dynamics that experience low-frequency (1–3 Hz), high-frequency (10–50 Hz), and large-magnitude (2,000–3,000 microstrain) (Fritton et al., 2000). Whole-body vibration (WBV) has been recently introduced as a bone stimulation therapy (12.6 Hz for 30 s with 1-min rest for 4 times) with hypoxic stimuli (16.1% FiO_2_) also showed improvement in bone mineral density (BMD) after 18 weeks (Camacho-Cardenosa et al., 2019).

Osteocyte cell death with age is one of the major factors for the onset of osteoporosis. Age-related osteoporosis is closely associated with a low level of autophagic activity, which was also shown in apoptotic osteocytes. There are several treatments for osteoporosis, reducing bone resorption, that are based on the administration of oral bisphosphonates (Fosamax, Boniva), intravenous bisphosphonates (Zoledronate, Pamidronate), Cathepsin K inhibitors (Odanacatib), and Anti-RANKL antibody therapy (denosumab) (Merlotti et al., 2007; Lewiecki 2010; Eriksen et al., 2014; Suen and Qin 2016; Tanaka et al., 2017; Lu et al., 2018; Galvano et al., 2019). Osteoblast-targeted hormone replacement therapy is also widely used, including estrogen receptor (Raloxifene) and parathyroid hormone peptide (teriparatide, abaloparatide), however, these therapies also affect osteocytes (Deal et al., 2005; Bonewald 2017; Leder 2017). *In vivo* studies have confirmed that sclerostin monoclonal antibody (Scl-Ab) treatment induced bone formation, mass, and strength (Yao et al., 2016). Scl-Ab products are commercially available including Romosozumab (AMG 785, CDP-785), Blosozumab, and BSP804 (McClung 2017; Morrell et al., 2021). These antibody-based treatments are widely used for reducing fracture risk arising from various health conditions, including osteoporosis as well as post-menopause (Ominsky et al., 2011; MacNabb et al., 2016). Furthermore, a bispecific antibody for sclerostin and DKK1 has been shown to have synergistic effects for bone formation compared with monotherapies (Florio et al., 2016). These approaches try to inhibit the secretory signaling molecules produced by osteocytes that antagonize the Wnt-signaling pathway in the osteoblast lineage, affecting the anabolic bone formation.

Although these treatments are widely used, the long-term safety and efficacy have to be taken into consideration. The most commonly used treatment for osteoporosis is based on bisphosphonates (Drake et al., 2008). These are effective and safe treatments with persistent benefit even after taking a break from the treatment, however, there are no clear guidelines for “drug holiday” (Diab and Watts 2013). The United States Food and Drug Administration (FDA) proposed a reevaluation of continuing bisphosphonate therapy after 3–5 years, showing a small decrease in BMD without higher fracture risk (Whitaker et al., 2012). In contrast to the prolonged half-lives of bisphosphonates, anti-RANKL (denosumab) shows reduced efficacy after treatment discontinuation (Bone et al., 2011). The anti-sclerostin treatment with romosozumab showed a decrease in BMD after discontinuation followed by 2-years treatment (McClung et al., 2018). Similarly, blosozumab treatment showed a decline in BMD in both the femoral neck and the lumbar spine after the discontinuation suggesting there is an increased risk of fracture (Recknor et al., 2015). The treatment options are summarized in Table 3.

**TABLE 3:** Treatment options for osteocyte-related diseases.

Treatment	Therapeutics	References
Antibody treatment	Sclerostin monoclonal antibody	(McClung 2017; Morrell et al., 2021)
	Romosozumab (AMG 785, CDP-785), Blosozumab, and BSP804	
	DKK1 antibody (BHQ880, DKN-01)	
Bisphosphonates	Oral bisphosphonates (Fosamax, Boniva), intravenous bisphosphonates (Zoledronate, Pamidronate)	(Merlotti et al., 2007; Lewiecki 2010; Eriksen et al., 2014)
Anti-bone resorption	Cathepsin K inhibitors (Odanacatib), and Anti-RANKL (denosumab)	(Tanaka et al., 2017; Lu et al., 2018; Galvano et al., 2019)
Hormone replacement therapy	Estrogen receptor (Raloxifene) and parathyroid hormone peptide (teriparatide, abaloparatide)	(Deal et al., 2005; Leder, 2017)
Non-invasive, painless mechanotherapy	Low-intensity pulsed ultrasound (LIPUS), vibration therapy, whole-body vibration therapy	(Fritton et al., 2000; Thompson et al., 2014; Thompson et al., 2016; Camacho-Cardenosa et al., 2019; Jiang et al., 2019)

Osteogenesis imperfect (OI) is a congenital disease that exhibits brittle bone (Basel and Steiner 2009). This disorder is caused by alterations in type I collagen that was previously known to be associated with osteoblast activities (Wenstrup et al., 1990). As the type I collagen is the predominant ECM protein, its dysregulation influences bone mineralization, leading to the impairment of local-acting growth factors such as TGF-β (Morello 2018). A recent study revealed that the osteocyte transcriptome was dysregulated in OI mice including Wnt/β-catenin and TGF-β signaling pathways (Zimmerman et al., 2019). TGF-β is a crucial factor to regulate bone formation and bone resorption for maintaining bone mass. However, excessive activation of the TGF-β signaling pathway found in OI increases high bone turnover and low bone mass (Lim et al., 2017). This continuous activation of TGF-β signaling may disrupt osteoblast functions while increasing osteocyte density. Increased TGF-β signaling can be diminished by TGF-β neutralizing antibody (ID11) treatment leading to improved bone mass by decreasing osteoblast and osteoclast numbers while normalizing the osteocyte density. The exact mechanism of impaired TGF-β signaling in OI is not fully understood, but possibly through impaired binding of small leucine-rich proteoglycans (e.g., decorin) to TGF-β in collagen fibrils. OI mouse model showed abnormalities of type I collagen expression showing abnormal osteocyte phenotype with impaired dendritic formation. Impaired osteocyte phenotype may contribute to their functional roles by interrupting the cell–matrix interaction. As a consequence, osteocytes may increase osteoblast activities towards bone formation after detecting a defective matrix, possibly for the restoration process. The osteocyte transcriptome sequencing of OI compared to wild-type control mouse models demonstrated the differential expression of dysregulated collagen fibril organization, but also impaired osteocyte dendritic formation, ECM compositions, and integrin-mediated signaling (Zimmerman et al., 2019). This observation supports the role of impaired cell–matrix interaction promoting dysregulated dendritic formation and leading to changes in functional roles. Interestingly, the Wnt signaling pathway in osteocytes was also affected in OI mice as gene levels for Wnt ligands were significantly increased, however, the exact mechanism of Wnt upregulation in OI remains unclear (Fahiminiya et al., 2013). *In vivo* studies with the conditional Wnt inactivation in osteocytes showed increased bone fragility and low bone mass as a result of altered Wnt1 production (Joeng et al., 2017). Like osteoporotic therapeutics, anti-resorptive (e.g., cathepsin K inhibitors and Anti-RANKLtherapies) and bone anabolic treatments (e.g., Sci-Ab and PTH) are commonly used for OI patients (Drake et al., 2008; Morello 2018).

Apart from bone-related diseases, there is more evidence emerging that osteocytes are also associated with other diseases, facilitated *via* secretion of the FGF23 hormone (Guo and Yuan 2015). It was reported that highly elevated circulating FGF23 is closely associated with kidney dysfunction, and this was also linked to heart failures such as left ventricular hypertrophy and vascular calcification (Faul et al., 2011; Desjardins et al., 2012). Furthermore, FGF23 was linked to chronic hypophosphatemia, caused by impaired mineralization of the bone matrix leading to bone fragility (Murali et al., 2016). Circulating FGF23 controls serum phosphate levels, by suppressing reabsorption in the kidney, and excess FGF23 causes hypophosphatemia diseases. Hypophosphatemia with high levels of FGF23, can be treated with a monoclonal FGF23 antibody (anti-FGF23), for example, burosumab, which was recently approved by the FDA to stabilize serum phosphate levels. The alternative medication for hypophosphatemia is a combination of active vitamin D and phosphate salts, however, this treatment often leads to kidney failure (Kinoshita and Fukumoto 2018; Barratt et al., 2021).

## Discussion

Once considered inactive cells, the osteocytes are now attributed to have crucial roles in the overall bone remodeling process, local microenvironment regulation and systemic interactions with other organs. The tightly regulated bone homeostasis becomes dysregulated as we age and with reduced mechanical stimulation, shifting the balance towards more bone resorption, leading to bone loss diseases such as osteopenia and osteoporosis, which increases fracture risk. As we move towards a more aging society, both intrinsic and extrinsic factors accelerate pathological signaling pathways causing disorders. Intrinsic factors (e.g., genetics, hormones, vasculature) and extrinsic factors (e.g., nutrition, physical activity, medications) are associated with the mechanisms that maintain healthy bone (Demontiero et al., 2012).

When the mechanosensitivity of osteocytes was first demonstrated, understanding the underlying modes of detection, the osteocyte-induced mechanotransduction pathways, and the functional outcomes for bone metabolism became significant research focuses in the field (Iolascon et al., 2013). The long life span of osteocytes (up to 25 years) and their important role in regulating the continuous coordinated cycle of bone formation and resorption and in the repair of bone damage makes them an ideal target for therapeutics. However, bone homeostasis is a complicated system involving multiple cell types that are signaling and coordinating each other. Until now, most studies on bone cells have focused on the more accessible effector cells, osteoblasts, and osteoclasts as compared to osteocytes (Liedert et al., 2005). The inaccessible location of osteocytes buried within the hydroxyapatite matrix, makes their visualization challenging, so studies have largely focused on *in vitro* cellular models to understand the mechanistic pathways that respond to mechanical stimuli.

Although *in vivo* studies provide more physiologically relevant outcomes, the various biological effects within more complex tissue responses are challenging to dissect and to attribute specific cellular roles given the complex microstructural organization of bone is hard to mimic. Targeting simplified approaches, focused on osteocyte-elicited mechanotransduction on 2D plastic or 2.5D using collagen coating, the *in vivo* three-dimensionality has been largely neglected in this field. Only recently, commercially available natural (e.g., collagen and fibrin), synthetic (e.g., polyethylene glycol hydrogels), and both animal and plant-derived (e.g., matrigel, gelatin, and alginate) matrices were used to construct 3D *in vitro* models (Langhans 2018; Zhang et al., 2019c; Aziz et al., 2020). The inorganic component, hydroxyapatite, is available as a ceramic composite with tricalcium/biphasic calcium phosphate (Boukhechba et al., 2009). Hydroxyapatite is widely used for coatings on metallic implants, bone fillings, and injectable bone substitutes (Ramesh et al., 2018). Alternative synthetic material, polystyrene is also available, and is tunable for various parameters such as pore sizes and thickness (Spatz et al., 2015). Direct cell-free bone tissue also becomes an option that represents the natural milieu, but again the mechanical properties are difficult to tune (Lyons et al., 2010; Li et al., 2019b).

Despite their inherent advantages, none of the cell models recapitulates the 3D dendritic morphology observed *in vivo*, indicating more ideal matrices need to be developed. This is especially important for osteocyte mechanotransduction studies, as the dendritic morphology is now considered an important mechanotransducer. Without providing an ideal microenvironment, this is not only limiting the morphology but also cellular responses, where better understanding of osteocyte mechanotransduction will provide significant opportunities for developing novel therapeutics for bone-related diseases.

The currently available treatments for bone disorders either target osteoclastic activity or osteoblastic activity (Rochefort 2014). Despite osteocytes abundance and their instrumental role in regulating bone metabolism, osteocyte-targeted treatments are not readily available. There are, however, some indirectly targeting antibody-based treatments to osteocyte-secreted molecules such as the recently FDA-approved sclerostin monoclonal antibody treatment for osteoporosis, promoting bone formation (Shakeri and Adanty 2020). Maintaining osteocyte viability is now considered one of the most important factors to maintain healthy bone (Bonewald 2017; Ru and Wang 2020). Aging, in particular, accelerates osteocyte apoptosis, resulting in fewer secretory factors, less bone matrix remodeling, and lower responsiveness to mechanical stimulation leading to impaired osteocyte functional roles in bone. Therefore, the development of novel osteocyte-specific therapeutics would be ideal to target osteocyte functions and signaling pathways including mechanisms to prevent apoptosis. Many of these pathways are still lacking a detailed understanding of, while others are more generic signaling pathways, such as the Wnt/β-catenin and TGF-β1 signaling, which are expressed in other cell types making therapies more challenging and less targeted (Janssens et al., 2005; Rys et al., 2016). In addition, the extra-skeletal roles of osteocytes in regulating distant organs, such as kidneys, heart, and parathyroid through secreted signaling molecules provides opportunities to target the associated dysfunctions in these organs through osteocyte manipulation.

The focus of this review was to highlight the fundamental role of osteocytes, the most mechanosensitive cells of the bone, by revealing how these cells detect mechanical stimuli through various mechanosensors and the proposed mechanotransduction pathways driving the functional responses that fundamentally affect bone metabolism. However, much detail around these mechanoresponsive pathways in osteocytes is still lacking. Therefore a greater understanding of these mechanisms will help us to identify more effective treatments for both chronic bone loss diseases such as osteoporosis as well as other genetic diseases affecting bone metabolism. This will also enable researchers to unravel, how these master regulators contribute to their important extraskeletal roles.
